# Absorbed Radiation Dose in Radiosensitive Organs Using 64- and 320-Row Multidetector Computed Tomography: A Comparative Study

**DOI:** 10.1155/2014/305942

**Published:** 2014-08-06

**Authors:** Atif N. Khan, Waqas Shuaib, Boris Nikolic, Mohammad K. Khan, Jian Kang, Faisal Khosa

**Affiliations:** ^1^Department of Radiology, Beth Israel Deaconess Medical Center, Harvard Medical School, Boston, MA 02215, USA; ^2^Department of Radiology and Imaging Sciences, Emory University Hospital, Atlanta, GA 30308, USA; ^3^Department of Radiology, Albert Einstein Medical Center, Philadelphia, PA 19141, USA

## Abstract

*Aim*. To determine absorbed radiation dose (ARD) in radiosensitive organs during prospective and full phase dose modulation using ECG-gated MDCTA scanner under 64- and 320-row detector modes. *Methods*. Female phantom was used to measure organ radiation dose. Five DP-3 radiation detectors were used to measure ARD to lungs, breast, and thyroid using the Aquilion ONE scanner in 64- and 320-row modes using both prospective and dose modulation in full phase acquisition. Five measurements were made using three tube voltages: 100, 120, and 135 kVp at 400 mA at heart rate (HR) of 60 and 75 bpm for each protocol. Mean acquisition was recorded in milligrays (mGy). *Results*. Mean ARD was less for 320-row versus 64-row mode for each imaging protocol. Prospective EKG-gated imaging protocol resulted in a statistically lower ARD using 320-row versus 64-row modes for midbreast (6.728 versus 19.687 mGy, *P* < 0.001), lung (6.102 versus 21.841 mGy, *P* < 0.001), and thyroid gland (0.208 versus 0.913 mGy; *P* < 0.001). Retrospective imaging using 320- versus 64-row modes showed lower ARD for midbreast (10.839 versus 43.169 mGy, *P* < 0.001), lung (8.848 versus 47.877 mGy, *P* < 0.001), and thyroid gland (0.057 versus 2.091 mGy; *P* < 0.001). ARD reduction was observed at lower kVp and heart rate. *Conclusions*. Dose reduction to radiosensitive organs is achieved using 320-row compared to 64-row modes for both prospective and retrospective gating, whereas 64-row mode is equivalent to the same model 64-row MDCT scanner.

## 1. Introduction

Multidetector computed tomography angiography (MDCTA) is uniquely suited to study cardiac anatomy and coronary artery disease (CAD) in a noninvasive manner and may even provide additional prognostic information to the baseline risk stratification [1]. The high sensitivity (85%–95%) and specificities (83–90%) of the newer generation scanners have already been documented [2, 3]. The high negative predictive value to rule out coronary artery disease (CAD) has prompted current European Society of Cardiology guidelines on management of stable angina to recommend MDCTA for patients with a low pretest probability of CAD and inconclusive stress testing [4].

The advent of 64- and 320-row MDCT have further improved the volume coverage, *z*-axis resolution, and scanning speed which has resulted in an increase in the number of potential applications of this technology and also the average scanned volume per examination. The new 320-row MDCT also produces better image quality as compared to the current 64-row MDCT [5]. The radiation dose associated with MDCT remains the single most significant concern in the context of its wider acceptability as a screening and diagnostic test [6, 7]. As MDCT is considered a major source of ionizing radiation in medicine, further in depth study of radiation exposure with these new scanners is therefore paramount [8, 9].

Amongst the various parameters which affect and account for the amount of radiation required during MDCTA examination are maximum body mass index (BMI), tube voltage (kVp), retrospective versus prospective image acquisition, volume scan length, heart rate (HR), and tube current time product (mAs) [10–17]. In addition, the effect of kVp variation on radiation dose depends on the type of CT scanner due to individualized internal scanner filtration and geometry. Also, results of kVp variations have generally been evaluated for the computed tomography dose index (CTDI) and/or CTDI water (CTDI_w_) but not as they pertain to specific organ doses [10, 16]. Reduction of kVp from 135 to 120 or 120 to 100 during cardiac imaging has also been shown to produce adequate image quality [5].

The objectives of our study were to measure and compare the ARD in radiosensitive organs (breast, lung, and thyroid) during MDCTA under the 64- and 320-row modes of operation to evaluate the effect of changes in HR and kVp on the ARD in these organs. Prior publications have emphasized the use of direct organ detectors for accurate measurement of ARD instead of the Monte Carlo System [18, 19].

## 2. Material and Methods

IRB approval was waived for this experimental study, which was performed on anthropomorphic phantom and did not involve patients or animals. All images were acquired with Toshiba Aquilion ONE MDCT scanner.

### 2.1. Phantom and Detectors

**Figure 1:**
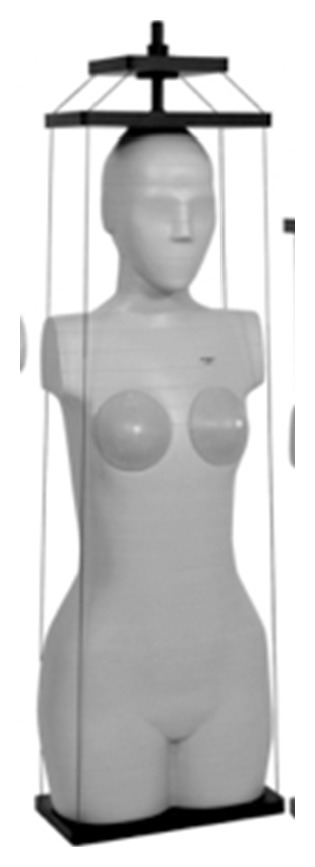
CIRS ATOM female phantom (Model number 702-D).

**Figure 2:**
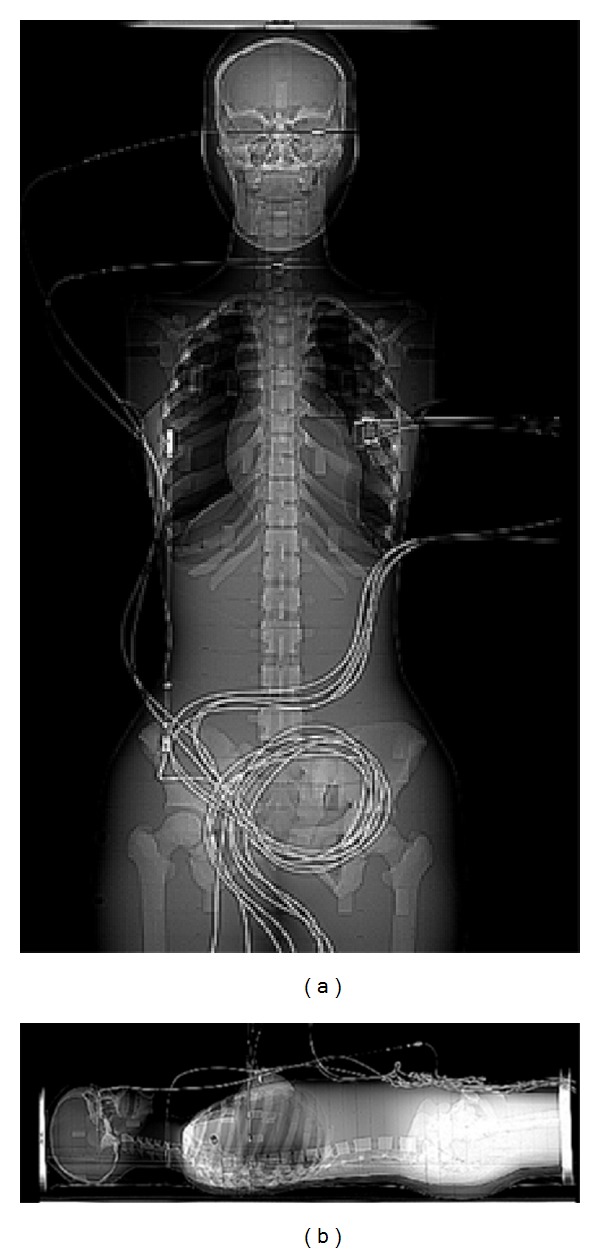
Phantom in supine and lateral view with detectors location.

The CIRS ATOM female phantom (Model number 702-D, Computed Imaging Reference Systems Inc., Norfolk, VA) is an anthropomorphic, cross sectional dosimetry phantom designed to measure organ radiation dose (Figure 1). The ATOM female phantom is manufactured with 38 slabs, 25 mm in thickness (each). It is important to mention that female phantom was used, specifically, to determine the absorbed radiation dose to the breast. Apart from the breasts, the female is equivalent to a small male phantom making it the ideal phantom to use. The phantom slabs are tightly bound together when in use for dosimetry measurements (Figure 1) with four strings, resulting in minimal interfaces between the slabs when viewed as a scout (Figure 2). The phantom provides predetermined locations specific to 21 internal body organs. Five custom made DP-3 solid state detectors (RTI Electronics AB, Molndal, Sweden) were used to measure absorbed radiation dose (ARD) to lungs, breast, and thyroid using different scan protocols on the Aquilion ONE scanner. The DP-3 detectors had been previously calibrated by RTI for measurements matched to the primary beam quality of the CT scanners. The protocols employed were designed to quantify radiation doses for prospective gated and dose modulation coronary angiography using MDCT. Three DP-3 detectors were placed in section 16 at locations 72, 73, and 75 representing the anterior, middle, and posterior locations in upper lobe of left lung, respectively. One DP-3 dose detector was placed in the right breast corresponding to the midbreast. One DP-3 detector was placed in section 10, location 26 representing the left lobe of the thyroid gland (Figures 2(a) and 2(b)). The output signals from these five detector probes were fed to the Barracuda Electrometer (RTI Electronics) equipped with multiple electrometer modules to accommodate concurrent data collection. The dosimeter information is in turn fed into a laptop computer running “Ocean” Software (RTI Electronics) for display and storage.

### 2.2. CT Imaging Protocols

All scans were performed with the Aquilion ONE 320-MDCT scanner under two different scan modes (64-row versus 320-row). The Aquilion ONE MDCT scanner is configured to operate as a 320-row MDCT and a 64-row MDCT. Under the 64-row mode of operation, the Aquilion ONE behaves essentially the same as the Aquilion 64-row MDCT. The anthropomorphic phantom was scanned in a single continuous session using both modes for prospective gated and dose modulated coronary MDCTA. The phantom was placed in supine position with phantom head entering the CT gantry first. Scout images were taken in anteroposterior and lateral direction to ensure the same volume coverage so data acquisition could be standardized for all measurements. All data were collected using the same 220 M (medium acquisition FOV) display field of view (D-FOV) and 160 mm volume scan length (VSL). The display field of view (D-FOV) is part of the reconstruction algorithm that sets the display size, pixel size, and display characteristics and volume scan length (VSL) is the area covered by the scanner along the *z*-axis. The mean of five radiation dose measurements for each organ at each HR and kVp was converted into the effective organ dose by multiplying it with the appropriate tissue-weighting factor for each organ [18, 19].

### 2.3. Coronary CT Angiography

MDCTA protocols (prospective gating) were studied at three different tube voltages 100, 120, and 135 kVp with a constant 400 mA tube current simulating two different heart rates at 60 and 75 beats per minute (bpm) with 64-row and 320-row modes. The volume scan length for each scan extended from one centimeter below the carina to the base of the heart.

In 64-row mode, prospectively gated MDCTA technique images heart with a step and shoot helical method with images captured every alternate heart beat. Gantry rotation time was 0.35 msec with 8.1 msec total scan time with a pitch of 17.1. A volume scan length of 160 mm and D-FOV of 220 mm were used to standardize all measurements. At 75 bpm, the Aquilion ONE in 64-row mode operates differently and acquires images of the heart with a dose modulation technique of 100 mA tube current during 30–90% of the R-R interval with a peak mA of 400 at 75% of the R-R interval. The table's pitch changes to 12.1 corresponding to 0.197 with an increased total scan time of 10.4 msec. Again, five datasets were taken and recorded for both heart rates using three different tube voltages (average of 5 was taken and recorded as the actual measurement).

The 320-row mode provides two different techniques for cardiac imaging: a prospective CTA and a second complete functional analysis with dose modulation. Under the 320-row mode, a total of 320 rows of detectors are available with a total collimator width of (320 × 0.5 mm) = 160 mm per rotation for data acquisition enabling the scanner to image the entire heart in a single gantry rotation (0.35 msec). The prospective CTA technique images the heart during 65–85% of the R-R interval with a slice thickness of 0.5 mm. A tube current of 400 mA, D-FOV of 220 mm medium, and VSL of 160 mm was used to take all measurements while utilizing three different tube voltages (100, 120, and 135 kVp) at two different heart rates (60 and 75 bpm).

The 64-row mode automatically selects modulation technique at a higher heart rate of 75 bpm. Tube modulation technique was also studied using the 320-row mode. The basic concept is almost the same with a baseline current of 100 mA during a complete R-R interval with a peak mA of 400 at 75% of the R-R interval. The CTA protocol with three different tube voltages at both heart rates was measured and recorded.

### 2.4. Statistical Analysis

Two-sample *t* tests were performed to detect the difference in ARD at breast, lung, and thyroid between the 320-row MDCT and the 64-row MDCT for each of the imaging protocols. In a subanalysis, linear mixed effects models were fitted to study both effects of tube voltage and heart rate variation on ARD, respectively, while adjusting for all other imaging factors (imaging protocols, scanners, etc.). In each model, a random intercept is specified to model random effects for multiple measurements. Wald tests have been used to determine the significance of effects for each factor.

## 3. Results

Mean ARD at 320-row versus 64-row modes were: midbreast (6.728 versus 19.687 mGy, *P* < 0.001), lung (6.102 versus 21.841 mGy, *P* < 0.001), and thyroid gland (0.208 versus 0.913 mGy; *P* < 0.001) (Table 1). Similarly with the dose modulation protocol, the ARD in the organs for the 320-row versus 64-row modes were as follows: midbreast (10.839 versus 43.169 mGy, *P* < 0.001), lung (8.848 versus 47.877 mGy, *P* < 0.001), and thyroid gland (0.057 versus 2.091 mGy; *P* < 0.001) (Table 3). The 320-row mode showed a significantly lower ARD at all detector's locations when compared to the 64-row mode equivalent for both imaging protocols.

In a subanalysis, linear mixed effects models were fitted to study the effects of tube voltage and heart rate variation on ARD while adjusting for all other imaging factors (Tables 2 and 4). In our study, the reduction of kVp from 135 to 100 kVp for prospective ECG gated MDCTA performed on 64-row mode and 320-row mode demonstrated a significant radiation dose reduction of 51.72% and 50.2%, respectively. However, it was noticed that a similar significant effect was not observed with a 120 to 100 kVp reduction in tube voltage. Similarly, the influence of variation in heart rate on ARD using prospective imaging methods was studied (Table 5). A similar trend was seen with dose modulation imaging for 64-row versus 320-row modes showing a reduction of 51.92% and 48.72% in ARD, respectively (Table 6). At a lower heart rate of 65 bpm, the 64-row mode delivered 63.1% less radiation dose while the 320-row mode delivered 43.82% less dose compared to doses delivered by each mode at 75 bpm.

## 4. Discussion

In our study, we measured the ARD with prospective gating as it delivers the lowest dose for assessment of coronary artery disease; similarly, we used DP3 detectors for acquisition of ARD measurements which are characterized by increased ease of use compared to thermoluminescent dosimeters (TLD)—the prior gold standard for point radiation dose measurement. In our study, ARD was measured for the most radiosensitive organs that are within the primary radiation beam (lung and breast). A small amount of osseous material (bone marrow) does lie within the primary beam and is, therefore, subjected to some degree of stochastic radiation. Thus, ARD in the sternum is well approximated by the chest wall measurement. Other organs within the primary beam such as neural, cardiac, and esophageal tissue are relatively radioresistant. Additional dose measurements addressing these anatomic structures were, therefore, not conducted in this study.

The differences seen above indicate a higher ARD with 64-row mode in radiosensitive organs in the scan field and attests to a greater radiation safety profile for the 320-row mode as studied on a standardized phantom. Our study supports the use of tube voltage and heart rate reduction to decrease ARD and is in synchrony with Matsubara et al. who obtained similar results on phantom studies with 64-row CT angiography [18]. Litmanovich et al. [20] also found better ARD profile in 64-row MDCT phantom study by using imaging protocols with lower tube voltage which was in consensus with an earlier study by Hurwitz et al. [21, 22]. Our study adds to the literature by documenting similar trend of kVp and HR variation on ARD seen in radiosensitive organs when using 320-row mode. Also, it advocates better radiation reduction of the 320-row mode for these radiosensitive organs when compared to the 64-row mode. Again, 64-row mode is similar to 64-row MDCT scanner in functionality.

Our data along with those of others also indicate that occurrence of deterministic skin effects secondary to MDCTA with the dual source 64- and 320-detector-row modes is fundamentally inconceivable, even in the setting of performance of multiple studies in a short time span in the same individual as deterministic effects have a threshold between 2,000 and 20,000 mGy, depending on the severity of the radiation damage. 

*Limitations*. Our study has a few limitations. First, only one body type anthropomorphic phantom was used. Patients with higher BMIs may receive ARDS that deviate from our results due to increased scatter radiation and the necessity to potentially increase tube current and voltage to obtain adequate image quality. ARD measured in lung and breast is a local dose and does not represent the equivalent organ dose.

The actual doses for any individual will vary from patient to patient, depending on tube current (mA) setting, heart rate, *z*-axis coverage, and body habitus. For prospective gating used in this study, absorbed organ doses are linearly proportional to the mA levels. This allows for easy calculations of different mA levels and makes additional radiation dose measurements unnecessary. Other scanning protocols, on the other hand, such as tube modulation would have a more complex effect on ARD and necessitate additional measurements. Finally, there is a lack of radiation data correlation to image quality or diagnostic accuracy assessment. The aim of this paper was to assess ARD through the comparison of 64-row and 320-row MDCTs as well as differing voltages. Because the anthropomorphic phantoms do not have actual cardiopulmonary structures, only the ARD could be measured while image quality cannot be assessed.

## 5. Conclusion

Coronary CT angiography can be performed with 320-row mode with much less ARD to radiosensitive organs as compared to 64-row mode at Aquilion ONE. Use of appropriate tube voltage (kVp) and heart rate control can further reduce the radiation dose to radiosensitive organs.

## Figures and Tables

**Table 1:** Comparison of Absorbed Radiation Dose (ARD) (mGy) for radiosensitive organs (Lung, Breast and Thyroid) between prospective imaging at 320-row MDCT versus prospective/step & shoot method at 64-row MDCT adjusting the effects of tube voltages and heart rates.

Locations	320-row mode	64-row mode	*P* value
Left Lung	6.102	21.841	<0.001
Left Breast	6.728	19.687	<0.001
Thyroid (Left lobe-26)	0.208	0.913	<0.001

**Table 2:** The effect of tube voltage on ARD (mGy) for prospective EKG gated/step & shoot method at both 64-row and 320-row protocols.

Protocols	kVp 100	kVp 120	*P* value
320-row mode	10.98	17.21	0.087
64-row mode	14.47	22.30	0.147

Protocols	kVp 100	kVp 135	*P* value

320-row mode	10.98	22.09	<0.05
64-row mode	14.47	29.97	<0.05

**Table 3:** Comparison of ARD (mGy) for radiosensitive organs (Lung, Breast and Thyroid) between dose modulation imaging at 320-row MDCT versus dose modulation method at 64-row MDCT.

Locations	320-row scanner	64-row scanner	*P* value
Left Lung	8.848	47.88	<0.001
Left Breast	10.84	43.17	<0.001
Thyroid (Left lobe-26)	0.057	2.091	<0.001

**Table 4:** The effect of tube voltage variation on ARD (mGy) for dose modulation protocol at both 64-row and 320-row modes.

Protocols	kVp 100	kVp 120	*P* value
320-row mode	17.57	27.15	0.072
64-row mode	38.76	61.34	0.131

Protocols	kVp 100	kVp 135	*P* value

320-row mode	17.574	34.26	<0.05
64-row mode	38.76	80.61	<0.05

**Table 5:** The effect of heart rate variation on the absorbed radiation dose (mGy) for both scanners.

Modes	60 bpm	75 bpm	*P*-value
320-row volume mode	15.50	27.59	<0.001
64-row helical mode	22.25	60.24	<0.001

**Table 6:** Percentage reduction in ARD with 320-row compared to 64-row mode.

Detector site	Percentage reduction in ARD (mGy) with 320 row mode MDCTA compared to 64-row mode MDCTA	
	Prospective ECG-gating protocol	Retrospective dose modulation
Left Lung	72%	81.52%
Left Mid-Breast	65.8%	74.8%
Breast Surface	64.53%	78.3%
Thyroid	77.2%	97.2%
